# Implementation of the e-SUS Primary Care system: Impact on the routine of Primary Health Care professionals*

**DOI:** 10.1590/1518-8345.4174.3447

**Published:** 2021-07-19

**Authors:** Tatiele Estefâni Schönholzer, Ione Carvalho Pinto, Fabiana Costa Machado Zacharias, Rodrigo André Cuevas Gaete, Maria Del Pilar Serrano-Gallardo

**Affiliations:** 1 Universidade de São Paulo, Escola de Enfermagem de Ribeirão Preto, PAHO/WHO Collaborating Centre for Nursing Research Development, Ribeirão Preto, SP, Brazil.; 2 Scholarship holder at the Coordenação de Aperfeiçoamento de Pessoal de Nível Superior (CAPES), Brazil.; 3 Scholarship holder at the Conselho Nacional de Desenvolvimento Científico e Tecnológico (CNPq), Brazil.; 4 Universidad Autónoma de Madrid, Faculdad de Medicina, Madrid, Spain.

**Keywords:** Health Policies, Primary Health Care, Health Information Systems, Electronic Health Records, Health Management, Unified Health System, Políticas de Saúde, Atenção Primária à Saúde, Sistema de Informação em Saúde, Registros Eletrônicos de Saúde, Gestão em Saúde: Sistema Único de Saúde, Políticas de Salud, Atención Primaria de Salud, Sistemas de Información en Salud, Registros Electrónicos de Salud, Gestión en Salud, Sistema Único de Salud

## Abstract

**Objective:**

to understand how the implementation of the e-SUS Primary Care system has been processed and its impact on the daily life of the health teams.

**Method:**

a qualitative research study, conducted in a municipality in the inland of the state of São Paulo with professionals who work in Primary Health Care and use the e-SUS Primary Care system as a work tool. Semi-structured interviews and thematic data analysis were used with Kotter’s three-phase approach.

**Results:**

a total of 17 professionals, nurses, physicians, dentists and community agents were interviewed. The implementation of e-SUS Primary Care and its impact on the daily life of health teams were understood in terms of mandatory implementation; weaknesses for implementation, such as absence of material resources and implicit imposition for the use of the system; fragile training for deployment and learning from experience.

**Conclusion:**

a harmful incentive process was observed, conducted from the perspective of institutional pressure, use of the system to justify the work performed and, on the other hand, there was the creation of collaborative learning mechanisms between the teams.

## Abstract (pt)

**Objetivo:**

compreender como se tem processado a implantação do sistema e-SUS Atenção Básica e seu impacto no cotidiano das equipes de saúde.

**Método:**

pesquisa qualitativa, realizada em um município do interior do estado de São Paulo com profissionais que atuam na Atenção Primária à Saúde e utilizam o sistema e-SUS Atenção Básica, como ferramenta de trabalho. Utilizaram-se entrevistas semiestruturadas e análise temática dos dados com abordagem trifásica de Kotter.

**Resultados:**

foram entrevistados 17 profissionais, enfermeiros, médicos, odontólogos e agentes comunitários. A implantação do e-SUS Atenção Básica e seu impacto no cotidiano das equipes de saúde foram compreendidos em termos da implantação obrigatória; fragilidades para a implantação, como ausência de disponibilização de recursos materiais e implícita imposição para uso do sistema; frágil capacitação para a implantação e aprendendo com a experiência.

**Conclusão:**

observou-se um processo de incentivo danoso, conduzido sob a perspectiva de pressão institucional, utilização do sistema para justificar o trabalho realizado e, em contrapartida, houve a criação de mecanismos de aprendizagem colaborativa entre as equipes.

## Abstract (es)

**Objetivo:**

comprender cómo fue el proceso de implantación del sistema de Atención Primaria e-SUS y su impacto en la vida diaria de los equipos de salud.

**Método:**

investigación cualitativa, realizada en una ciudad del interior del estado de San Pablo con profesionales que trabajan en Atención Primaria de la Salud y utilizan el sistema de Atención Básica e-SUS, como herramienta de trabajo. Se utilizaron entrevistas semiestructuradas y análisis temático de los datos con el enfoque de tres fases de Kotter.

**Resultados:**

se entrevistaron 17 profesionales, enfermeros, médicos, odontólogos y agentes comunitarios. La implantación de la Atención Primaria e-SUS y su impacto en la vida diaria de los equipos de salud se entendieron en términos de implantación obligatoria; debilidades para la implantación, como la ausencia de recursos materiales y la imposición implícita del uso del sistema; escasa capacitación para la implantación y aprendizaje de la experiencia.

**Conclusión:**

hubo un proceso de incentivo nocivo, realizado bajo la perspectiva de presión institucional, uso del sistema para justificar el trabajo realizado y, en contrapartida, se generaron de mecanismos de aprendizaje colaborativo entre los equipos.

## Introduction

In the health sector, information supports the planning, decision-making process and implementation of public policies; however, limitations related to the permanence of manual data records, difficulties in accessing computer equipment, and poor training of human resources can negatively impact on its operationalization^1^.

Lack of skill can be seen as a barrier in the daily lives of the professionals, when it comes to maintaining the quality of the data, either due to lack of skill or to time constraint^2^. These negative impacts can be minimized through the perception of the professionals about the quality factors of the information system and by the motivation to use it^3^. Since it is essential to pay attention to the interaction between the demands of the professional, the technology and the desired context^4^.

In health, changes are recognized as important; however, they are interspersed with challenges due to the administrative complexity of this sector and, for the implementation of any change be successful, it is necessary for health organizations to be able to manage such a process. There are aspects that can be cited as forbidding for implementing computerized information systems in Brazil, such as impasses during the implementation, difficulties in the preparation of the professionals, as well as the deficiency of the organizational structure^5^.

In this implementation process, elements such as vision, skills, encouragement, resources and action plan lead to a real change; however, the absence of one of these elements in the process can cause feelings such as anxiety, confusion and frustration^6^.

In Brazil, information is used as an instrument for the management of the Unified Health System (*Sistema Único de Saúde*, SUS), related to health surveillance, production monitoring and financial transfer, and may highlight administrative management and often distance itself from the needs of the health services^7^.

Until 2013, in Primary Health Care (PHC), the Basic Care Information System (*Sistema de Informação da Atenção Básica*, SIAB) was used, but there was no structure to respond to the needs of the population. Thus, a new strategy was designed to serve them, with the institution of e-SUS Primary Care (*e-SUS Atenção Básica*, e-SUS AB). This implementation brought changes both in the health services, such as new technologies, as well as in the ways of collecting, processing and using information in the care and management process, through e-SUS AB, focusing on local management, and the Health Information System for Primary Care (*Sistema de Informação da Atenção Básica*, SISAB), focusing on municipal, state and federal management^8^.

Currently, 72.8% of the Basic Health Units (BHUs) of the Brazilian municipalities have the e-SUS AB system implemented, with 43.9% using Simplified Data Collection (*Coleta de Dados Simplificada*, CDS) and 29% using the Electronic Citizen’s Medical Record (*Prontuário Eletrônico do Cidadão*, PEC)^9^.

In the implementation of information systems, structure and technical support are needed to support the process and, even when made available, obstacles can still occur at the local level. The criterion of the Ministry of Health to determine whether the BHUs use the system is sending data to the SISAB; however, there is no information or analysis about the real implementation scenario or about how the process was inserted and perceived by the professionals. Thus, this study aimed to understand the implementation process of the e-SUS AB system and its impact on the daily life of the health teams.

## Method

This is a qualitative research study, conducted in 2018 in a municipality with a population of approximately 25,000 inhabitants and a high Human Development Index (HDI) (between 0.7 and 0.8)^10^, in the inland of the state of São Paulo. The health network of the municipality is composed of seven teams of family health strategies, an emergency care service, a home care service, a specialized dental care center, a specialty outpatient clinic and a Psychosocial Care Center (*Centro de Atenção Psicossocial*, CAPS). The study scenario consisted of six family health strategies that had the e-SUS AB with PEC implemented, located in three peripheral and three central areas of the city.

The study population consisted of 17 health team professionals (nurses, community health agents, dentists, physicians and nursing technicians) with experience in using the e-SUS AB system for at least six months and who were open to dialogue, selected for convenience. The criterion was the repetition and saturation of the data to determine the number of interviews, and at least two professionals *per* category^11^.

The research was developed by the main author who had previous experience in collecting and analyzing qualitative data, not having interpersonal ties with the study participants. There was previous contact with the field and with the participants through visits, to present the project, conduct pilot interviews and schedule data collection. A semi-structured interview script was used for data collection, in a private environment provided by the BHU managers.

The interviews were estimated at up to 60 minutes, and were recorded on two audio devices simultaneously. The questions of the interviews addressed the implementation process of the e-SUS AB system. The interviews were fully transcribed, coded according to the sequence in which they were performed and identified, considering the location in which the BHUs were inserted, Central Area (*Zona Central*, ZC) and Peripheral Area (*Zona Periférica*, ZP), followed by professional category and ordinal number.

To process the data from the interviews, thematic analysis was used, contemplating the following stages: familiarization with the data; generation of initial codes; research, review, definition and name of the themes and production of the report^12^. After transcribed, with the help of the Google Docs text editor by means of the “voice typing” tool, the interviews were coded according to professional category and ordinal number and, from this, the reading of the transcribed material began with the insertion of comments recorded by the researcher, as the entries caught her attention.

Next, the initial codes were elaborated and, subsequently, the phase of grouping and/or separation of codes took place^12^. The initial codes were inserted in the Word text editor, on a blank page, to verify the possibility of groupings among them, always seeking to ensure correlation between the information. Later, the potential themes were transferred to a sheet of paper and, with the use of colored pens and adhesive papers, the list and review of the themes was built and, finally, the central theme was reached.

The management of change in health was used as a framework, which incorporates Kotter’s three-phase approach^8^. Kotter’s three-phase approach to change management is based on three distinct phases: creating a climate for change, engaging and empowering the organization, and implementing and sustaining the change. This health-driven model can be used from planning changes in the way to care for the patients to changes in the use of technologies to provide more safety and quality^8^.

During the analysis process, to confer credibility, consistency and confirmability, a discussion was held among the members of the research group, as well as feedback to the participants.

The research project was approved by the Research Ethics Committee of the Ribeirão Preto School of Nursing, University of São Paulo, with protocol number: 73772817.3.0000.5393, in compliance with the Guidelines and Regulatory Standards of Research Studies Involving Human Beings, approved by Resolution 466/12 of the National Health Council.

## Results

Four community health agents, four nurses, three physicians, four dentists and two nursing technicians participated in the study. Of these, 15 (88%) were female and 2 (22%) male, with a mean age of 35 years old, ranging from 29 to 51 years old, 12 (70%) had complete higher education and five (30%) had complete high school, being active in the BHUs from 1 to 17 years, with a mean of 6.7 years of work.

To analyze the data from the interviews, the coded material made it possible to observe, in the organization of the statements, that the themes evidenced refer to the implementation process. Thus, there are four categories, namely: “Mandatory implementation. ‘You have to implement’”; “Weaknesses for the implementation of the system”; “Fragile training to achieve implementation” and “Learning from the experience”.

### Mandatory implementation. “You have to implement”

In the PHC health units, institutional pressure to implement the e-SUS AB Strategy culminated in the implementation and mandatory use of the information system.

*[...] They said: From next month you need to use the e-SUS... And then they acted like this [...] (ZPENF03).*

*[...] They just spoke like that, putting fear: you have to do; have to learn, it was like that [...] (ZCDEN01).*

This pressure generated two segments, one positive, reflected as the implementation for the operation of the process of recording, sending, processing and returning the information to fulfill its purpose; and the negative, evidenced by the non-planning of the process that culminated in an accelerated deployment to meet the deadlines established for effective use and sending of data. The professionals who experienced this phase describe it in a negative manner.

*[...] I don’t remember the year anymore... but it went from one month to the next. And so, they left it to the last minute, so they presented it to us more or less... So in one month we placed everyone on the computer… and it was that fight, in a month we attacked [registered] the whole area, so we take turns here [...] (ZPACS04).*

*[...] There was not much time, we started talking and we had to run with the registrations, changing quickly like that, I think that there was a little lack of time [...] (ZPACS01).*

### Weaknesses for the implementation of the system

The lack of availability of material resources permeated the experience of the health professionals, in the phase of implementation and use of the e-SUS AB system.

*[...] The difficulty... at the beginning was the lack of Internet because, like that, there were days that I had Internet, days that I didn’t... but not today, today the Internet is difficult the day that there is no Internet [...] (ZPENF04).*

*[...] At the beginning there was a bit of a fight because there were two teams in the past, BHU A and B, so they argued in relation to the computer scale, that one was more than the other, then it made it difficult to pass the production [typing records] [...] (ZCENF02).*

Regarding the implementation process, another important point to highlight is that it did not come with the team’s engagement, regarding the importance of the Strategy and the e-SUS AB system, but with an implicit imposition for the professionals to start the registration.

*[...] You know that thing... You have to, you have to implement... Just like that, there is this time, then it seems that the person took advantage of this time not to pass [implement], and there was one month left to go: let’s do it, if not, we’ll lose the funding! [...] (ZPACS04).*

*[...] They said, well, you have to improve the number of visits. Such a place has a lot of service, such a place has less to compare units, you know? But just [...] (ZCMED01).*

### Fragile training to achieve implementation

The participants’ narratives about the lack of sufficient and adequate training, before or during the implementation process, show doubts about how to use the e-SUS AB system in all its potential, lack of understanding of the Strategy purpose, as well as its relevance to PHC. In addition, attempts at training were reported; however, they were identified as insufficient to solve doubts, creating an impasse to register data in the new system.

*[...] A guy came asking us not to be leaving the unit because he was going to be passing by to explain the e-SUS, and we stayed in the unit all day, all day... then he arrived... and we even questioned, that our schedule is from seven in the morning until four in the afternoon, he arrived here at 3:55 pm... Our service is on time we are here, then we want to leave. So as he arrived at 3:55 pm we just signed the sheet and left [...] (ZPACS04).*

*[...] We didn’t have any training, or anything, it was something very basic and they said: You have to do this, this and this. Then doubts were cleared throughout the day, as we were using it. However... I can’t do it, I still have doubts [...] (ZPENF03).*

The professionals exposed difficulties arising from this pseudo-training concomitant with the system updates. There have been updates since the implementation of the e-SUS AB 2.0 system until the time of the interviews (e-SUS AB 3.0). This, added to the lack of training, leaves the professionals imbued with doubts that accumulate with each version.

*[...] They called, explained... I think that not everyone was together, there was a nurse and assistant first and then the health agents... but, then, it was few times. Then it changed, then after it changed it didn’t explain to me either, so, the health agents end up having a lot of difficulty during the day to day [...] (ZPENF04).*

*[...] Because I think so, it looks like it (e-SUS AB) from time to time, I don’t know if it’s from month to month, there is a change. For the better I think [...] (ZPACS04).*

*[...] If the e-SUS, just like now, the sisprenatal will no longer exist, everything will be in the e-SUS, so I think they should invest more in this training, they are expanding the system [...] (ZCENF02).*

The recurring doubts generated insecurity and distrust in the dentists regarding the system.

*[...] I have [the appointments], except that everything is saved and every 30 days I print a report also of everything that was attended to. I like to archive (ZCDEN02).*

*[...] Usually the system crashes or the Internet fails. So I don’t trust 100%... So I prefer to have everything on paper written down. And I don’t throw away the one that was taken down, it’s filed with me. And luckily for me, because a short time ago, a survey of the procedures conducted was carried out and came... far below what I had accomplished. So one more reason for me to continue doing this situation of putting it on paper and on the record, as a way for me to protect myself [...] (ZPDEN04).*

In the absence of technical support for the training of new professionals and recurring updates, the nurse manager of the unit and some CHAs who had knowledge and skills with the use of technologies were added, the role of trainer.

*[...] I don’t know, I think that it’s very well played, so you know, the people who had to learn. So that a doctor enters, they don’t have the training to use the e-SUS, we, as a nurse, have to stay there: “look, do it like this”... no... it’s not a part that we use and it’s not even possible... our responsibility to be training doctors or other professionals [...] (ZPENF03).*

*[...] I feel that a period of the month comes when they, the agents, are under the pressure of having to throw the production, and the nurse is overwhelmed by having to keep asking. About nursing, and assistant, in short, I also feel because it makes it difficult, slows down her work [nurse], because the person doesn’t know how to use the computer part that much, it depends on another [...] (ZCMED01).*

### Learning from the experience

When they perceive themselves immersed in this process described above, associated with the use of the new system to carry out their work, the professionals developed learning mechanisms over time, whether individual or cooperative, within the same unit or across health units.

*[...] At first nobody knew anything about the e-SUS. We basically learned on our own. I had a colleague who worked with me who understood computers, we started to understand and teach other colleagues too (ZCACS02).*

*[...] We go asking each other here. We don’t have... there’s a guy to tell you the truth, who is called [name] and he’s [name of the BHU] a health agent, and they say that he likes this IT situation a lot and he goes finding some way out. So there was already a situation or two that I asked him (ZPDEN04).*

*[...] The person who explained to me how to use it was the previous doctor who was there. One day we were together here and she explained more or less how she uses it (ZPMED03).*

Unlike the old information system, the SIAB, the e-SUS AB system allows for the interaction of the professionals due to its interface, which made decentralization of work possible, reducing the responsibility for the production of the team, as well as the burden on the nursing professionals, who, in their great majority, assumed the role of gathering the care records of the team and registering with the SIAB.

*[...] We’re much more in touch. I find it easier. This SIAB we filled in the sheet here... a white sheet, the nurse would pass and stay... that was it, so, I think this one here we have more access to, than this SIAB [...] (ZCDEN02).*

*[...] You spent another day. You had to pass all the health agents, assistant, I think that each one now does their own, I think that this way... each one is responsible. Because wanting or not they end up being responsible for their production because it was not. So, I did or did not do it myself. And even I was going to answer, so I thought it helped a lot [...] (ZPENF04).*

*[...] It wasn’t us who fed the SIAB... we simply sent the production to the coordinator and they fed the SIAB. We never did this direct feeding. Got it? Then I think. It’s some... kind of control, but a more intimate situation with people, with eating. I thought it was cool [...] (ZPDEN04).*

Even using parallel learning mechanisms, it is noted that the system is not implemented and that the information is used as an element of care qualification, but rather as accountability/justification for the managers.

*[...] We have to make a reference that we are there, dealing with, what we are doing here. In a way some accountability, for... a financial organization and to know exactly what is being spent, how much it is necessary to contribute in the service. So I think it’s in that sense [...] (ZPDEN03).*

*[...] For health? No… for them there [government], for them there, for income generation, for us not? One control, one... how am I going to talk... Unit organization [...] (ZCTE011).*

*[...] For us... to be able to record the information we have about the patients... so I can have one... also in a quantitative, qualitative way of the things we do... in order to be registered, both issues of... social and geographical information, among other things and also procedures that we have... for the ministry to know how much they spend on all this and also... regarding information... of the population itself [...] (ZCENF01).*

## Discussion

In 2018, the municipality under study was in a situation with the PEC System in place, given the data from the Ministry of Health (Jan/2019), in addition to being among the municipalities in the region with the highest volume of data sent to the SISAB.

The climate of change, during the implementation of the system, sustained by the first Kotter’s phase, and generated feelings of disappointment and confusion among the professionals in the face of the failures that occurred during the process of acceptance and use of the system. These difficulties have already been identified in the implementation of other information systems, in Primary Care in Brazil^13^. In addition, the verticalization of policies and regulations^14^ can contradict actions at the local level. In this case, such verticalization was permeated with mandatory and punitive aspects that reverberate mainly in the municipal sphere^15^, influencing the change management process.

When it comes to change management, it is foreseen to set goals; however, just stipulating dates does not materialize processes. Lack of planning was evidenced when comparing the period of implementation in the municipality against the deadlines stipulated by the Ministry of Health to implement the e-SUS AB Strategy, initiated in August 2013, with its first deadline in July 2014, and the second deadline for December 2015, with an interruption in the transfer of funds scheduled from April 2016^8^^,^ 16.

With support from the second Kotter’s phase, the engagement and training of the professionals was evidenced by lack of ability to use the system due to the non-qualification of material resources that, in the perspective of the professionals, made the change process frustrating. In other settings, there was the same difficulty as for the qualification of the professionals for using the e-SUS AB system, such qualifications being determined as ineffective^17^^-^ 18.

Considering international implementation experiences, training positively impacts on the professionals’ experience, as well as on the knowledge to manage the implementation process and the transition between the systems^19^^-^ 20. In addition to this, a study shows that using the aspects of change management, during the implementation of an electronic medical record, improves digital transformation in the health service^21^.

In an attempt to sustain the changes, as proposed in the third Kotter’s phase, the professionals developed collaborative learning networks between team members and across the BHUs. Teamwork in health is considered as a way of relating within a group process, in which exchanges of knowledge and interests occur, forming a network of relationships among people^22^.

The learning mechanisms incorporated an exchange of knowledge between the professionals of the same team or in groups to share messages, being reported as fundamental for the use of the system and, consequently, for the development of the professionals’ work during the beginning of data sending to the SISAB. In another study, carried out in Minas Gerais, collaboration was also evidenced between colleagues with the use of a group in a multi-platform of instant messaging related to the e-SUS AB system^15^.

It is worth reflecting that, even though cooperation for learning is a positive point, there are concerns regarding what is passed on among the colleagues, the diversity of manners to record the understanding on the proposal of the system for managing and qualifying care. These factors can generate, respectively, inconsistencies and lack of integrity in the records entered by the professionals, in the electronic medical record, as well as affect the quality of the information^23^.

As a limitation of this study, the non-inclusion of the professionals on the commissions responsible for implementing it in the study scenario is highlighted, which would allow for an expanded understanding of this process. However, this study contributes as a management tool for the managerial bodies in the process of health change, such as the implementation of the e-SUS AB system and the impact on the daily lives of health teams.

## Conclusion

There was an implementation process initiated with feelings of confusion and disappointment. Absence of vision, skill and resources can be evidenced, in addition to a harmful incentive process, conducted under the perspective of institutional pressure associated with the possible absence of planning. On the other hand, from the experiences and to meet the needs of the lack of ability to use the system, collaborative learning mechanisms were created.

These shared experiences can be directed in more assertive actions by the managerial bodies, given the implementation of the e-SUS AB system, with the improvement of user qualification, ensuring the necessary recourses for the infrastructure and support provision, aiming to guarantee the quality of the system, of the information and of the health service.

The periodic assessment on the situation of the implementation and use of the system in Brazil is necessary because, in addition to being an emerging reality, it can assist in improving the policies to achieve the objectives of the e-SUS AB Strategy and its national coverage.
